# Woman and Chivalry

**Published:** 1914-08

**Authors:** 


### Woman and Chivalry.

Is it literally true that men are temperamentally different from what they were when knighthood was in flower, as someone has said?

If chivalry is gradually giving way to coarseness and brutishness in a way to make the chivalrous grieve; if men no longer feel it a pleasure as well as a duty to surrender a seat to a woman who is standing, to uncover their heads in the presence of femininity in the elevator or wherever else lifting the hat as a mark of respect, whose is the fault?

A writer in one of the current magazines discusses the changed attitude of men towards women as a result of changed conditions and environment since women began to invade the business and the professions as competitors of the sterner sex. In a former time, the man of average culture and refinement, even some of the coarser grained, esteemed it a pleasure and a privilege to make some personal sacrifice for a pretty woman or an elderly woman, and would feel amply repaid by even the mute "thank you" expressed by the faintest semblance of a smile. Now we are given to understand that men and women elbow each other with as little compunction as if both were of the same sex.

Well, whose fault is it? Is it the fault of the women who have been forced by necessity into the avenues of bread-winning—by the failure of their fathers and brothers to provide for their needs? Are the working women in the channels of trade and industry such from choice, as a rule? Would they be crowding the men in the wage-earning fields if grim necessity did not compel them to do so? Native gallantry should accord to the women of spirit the same chivalrous respect and consideration that may be given to their weaker and more dependent sisters; labor that is honorable should never be regarded as detracting from dignity or the respect due to those who perform it, whether from choice or necessity.

The young woman or the older sacrifices nothing of character or dignity or of natural charm by making herself useful, by asserting her independence of charity or unearned support, even though the exigencies of bread-winning may bring her into open competition with the male sex. The respect she challenges and maintains depends upon her deportment, not upon her wage-earning activities. The days of chivalry have not passed. The honor that is womanhood's due is in evidence as much today as in knighthood's day, though it be less volatile and less obtrusively spectacular; and it will continue to be as long as enlightened civilization makes for the nearer perfect man; as long as "gentleman" is synonymous with courtesy and consideration, and gentlewoman the proudest title we can give to our womankind.—*San Antonio Express.*

There is nothing new or startling in this statement, but, appearing at this time in a leading daily paper as its expression on the much discussed feminine movement, it is not without interest. Much confusion exists in the minds of most people in regard to the woman question. The "right to vote" is one thing, and "woman's rights," as interpreted by the feminist, is another. The extension of the franchise to women is simply a constitutional enactment, but to extend to them equal privileges, e. g., granting them the right of sexual selection in the same sense that men exercise this right is to change the mores of society in respect to our regard of the value of virtue. The one is an experiment in government as a question of expediency with strong probability of improving social conditions, while the other is an experiment in changing the elemental racial forces, which is fraught with great danger.

Chivalry is fostered on respect for woman and belief in her virtue. It finds its highest expression in protecting this virtue at any cost as a question of honor. And it finds its reward in the constancy and devotion of requited love. So long as virtue lasts and is held above price, so long will chivalry champion the cause of woman, no matter her creed or social position, and it will ever be her best and surest protection.

---

DR. H. S. ROBERTSON, of Oakhurst, Texas, left June 25th for New York, to spend a couple of months in post-graduate schools. While there he will do research work.

